# Recent advances in the study of reproductive function in pediatric patients with brain tumors

**DOI:** 10.3389/fped.2025.1625359

**Published:** 2025-07-10

**Authors:** Rui Zhang, Linlin Liu, Xinyu Dai, Yan Ju, Yanming Ren

**Affiliations:** ^1^ Department of Neurosurgery, West China Hospital, Sichuan University, Chengdu, China; ^2^ The First College of Clinical Medicine, Chongqing Medical University, Chongqing, China; ^3^ Department of Anesthesiology, The First Affiliated Hospital, Chongqing Medical University, Chongqing, China

**Keywords:** pediatric, brain tumors, neuroendocrinology, reproductive function, HPG axis

## Abstract

In recent years, pediatric brain tumors have emerged as one of the most common malignant tumors among children. The site of tumor growth and the selected treatment modalities can potentially have a profound and significant impact on the reproductive function of pediatric patients, which is intimately associated with the physical and psychological health of children during their developmental process. Current research studies have demonstrated that pediatric patients diagnosed with germ cell tumors, craniopharyngiomas, and medulloblastomas commonly present with reproductive dysfunction. Regrettably, in clinical practice, neurosurgeons frequently fail to allocate sufficient attention to this particular aspect. It is of critical and urgent necessity to explore and elucidate the alterations in reproductive function among pediatric patients with brain tumors, and subsequently institute essential protective measures. This article is dedicated to comprehensively reviewing the latest research advancements regarding the relationship between pediatric brain tumors and reproductive function, thereby providing a valuable reference for safeguarding the reproductive function of pediatric patients afflicted with brain tumors.

## 1 Introduction

Brain tumors are the most common solid tumors in children (1, 2). Based on the anatomical location, tumors can be classified into supratentorial tumors and infratentorial tumors. Supratentorial tumors are more frequently observed in children under the age of 3 and over 10 years old, whereas infratentorial tumors are more commonly encountered in patients within the age range of 4–10 years old (3). Meanwhile, it is worthy of note that patients of distinct ages exhibit a predisposition to different varieties of tumors. Younger children have a higher propensity for embryonal tumors like medulloblastomas or atypical teratoid/rhabdoid tumors, while older children are more susceptible to glial neuroglial tumors (3).

With advances in technology and treatment methods, the survival rates of children with brain tumors have significantly improved, leading to extended lifespans. However, this has brought greater attention to long-term neurological challenges such as impairments in motor function, sensation, speech, and other related issues. Among these complications, reproductive dysfunction—a concern for some cancer patients—remains one of the most often overlooked aspects of survivorship care.

Injury to the hypothalamus-pituitary-gonad (HPG) axis frequently occurs in patients, consequently exerting an adverse effect on reproductive function (4–8). Reproductive function is of vital significance for the physiological and psychological well-being of children during their growth. The impairment of reproductive function represents a substantial setback for patients and their families. Hence, it is essential to explore the alterations in reproductive function among pediatric patients with brain tumors and reinforce the protective strategies during treatment.

We searched the PubMed database using the keywords “Pediatric Brain Tumors and Reproductive Function” for literature published between 1989 and 2024, yielding a total of 206 articles. Subsequently, we applied our inclusion criteria: first, the study must focus on pediatric brain tumors; second, it must be related to the reproductive function of pediatric patients. All literature unrelated to the reproductive function of pediatric brain tumor patients was excluded. From the articles meeting the inclusion criteria, we further identified the most relevant studies, ultimately narrowing down the selection to 73 articles. This review aims to summarize the latest researches on reproductive function in pediatric patients with brain tumors, and it is anticipated that medical practitioners will attach significance to the preservation of the reproductive function of pediatric patients.

## 2 Causes of reproductive dysfunction in pediatric patients with brain tumors

Reproductive dysfunction in pediatric patients is primarily caused by abnormalities of the HPG axis. The tumor type, location, and treatment methods can all affect the HPG axis (9). Tumors may influence reproductive function by releasing specific hormones, such as gonadotropins (10). The tumor in proximity to the hypothalamus-pituitary region can directly impinge upon the function of the HPG axis due to its mass effect or invasive growth pattern (11).

**Figure 1:**
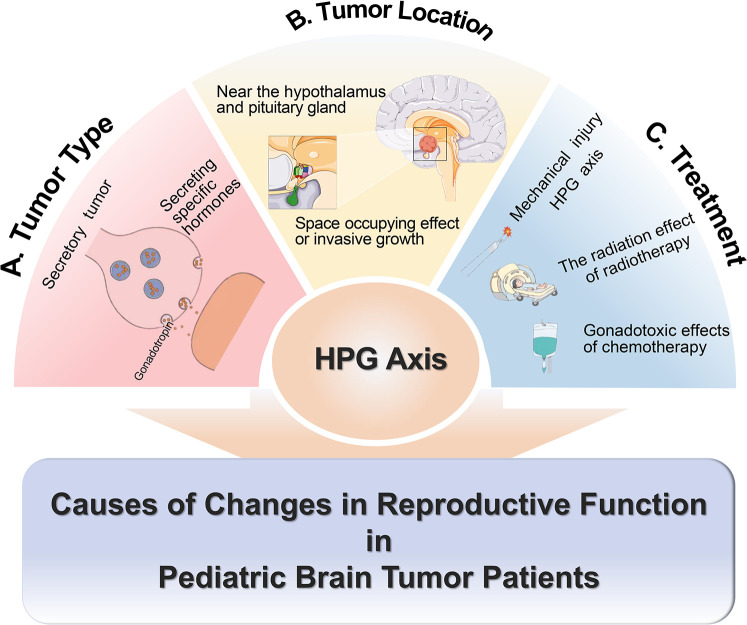
Causes of changes in reproductive function in pediatric patients with brain tumors.

Attention should also be directed towards reproductive dysfunction resulting from treatment processes. Diverse treatment regimens are selected for various types of pediatric brain tumors, causing varying extents of reproductive dysfunction. Most tumors require surgical removal. If the HPG axis is damaged by mechanical trauma during surgery, it may lead to irreversible damage to reproductive function. For patients undergoing radiotherapy and chemotherapy, the radiation from radiotherapy and gonadal toxicity of certain chemotherapy drugs can cause varying degrees of reproductive dysfunction. For example, in medulloblastoma patients, the impact of radiotherapy on reproductive function varies with gender, age, treated area, and radiation dose. Women and those who are going through puberty during radiotherapy have a higher risk of gonadal insufficiency (12). In the area of radiotherapy, gonadal radiation dose (GRDE) has a greater direct effect on reproductive function than hypothalamic radiation dose. GRDE >2 Gy is associated with gonadal insufficiency in all age groups, and for prepubertal patients, this association occurs at doses >1 Gy (12). While studies on radiation dose in the hypothalamus have shown that cranial irradiation with at least 18 Gy has been associated with central precocious puberty in childhood cancer survivors (13). The damage to gonads from chemotherapy mainly relates to high-risk chemotherapy regimens (9). High-risk chemotherapy includes either high-risk drugs (busulfan, melphalan, thiotepa, cisplatin, carboplatin) or combinations of multiple moderate-risk drugs (low-dose alkylating agents like cyclophosphamide, ifosfamide, or procarbazine; anthracyclines such as daunorubicin, doxorubicin, or mitoxantrone; and drugs like lomustine or carmustine) (9). This suggests that treatment should be individualized based on each patient's condition (Figure 1).

## 3 Reproductive function status in pediatric patients with common brain tumors

Accumulated evidence has demonstrated that pediatric brain tumors, including germ cell tumors, craniopharyngioma, and medulloblastoma, are capable of inducing reproductive dysfunction (4–8). Interestingly, certain patients exhibit the initiation or restoration of the HPG axis following treatment, suggesting that diverse tumor types, treatment modalities, and other elements can influence the extent of HPG axis impairment (4–8).

### 3.1 Patients with germ cell tumors

Intracranial germ cell tumors are rare and originate from the residual tissue of primordial germ cells, accounting for approximately 3%–5% of malignant tumors in pediatric brain tumors (14). The peak incidence of central nervous system (CNS) germ cell tumors is observed between 10 and 12 years of age, and the incidence rate in males is 3–4 times higher than that in females (15, 16). Germ cell tumors predominantly occur in the pineal and suprasellar regions (17). In comparison with the tumor situated in the pineal region (5-year survival rate is about 60%), the tumor in the suprasellar area has a less favorable prognosis (5-year survival rate is only 20%) (18).

Pediatric patients afflicted with germ cell tumors frequently manifest endocrine symptoms. Roughly 50.9% of such patients will endure impairment of the HPG axis, and approximately 10.7% display hypogonadotropic hypogonadism, especially when the tumors are located in the suprasellar region (19). This phenomenon might stem from the direct influence of tumors within this particular region. Hypogonadotropic hypogonadism, along with other endocrine manifestations, represents the principal symptoms that are correlated with a delayed diagnosis (20). In addition to the retarded sexual development resulting from hypogonadotropic hypogonadism, patients may also exhibit precocious puberty (21, 22). This is primarily attributed to the abnormal secretion of human chorionic gonadotropin (HCG) by tumor cells, which stimulates gonadal hormone secretion, inhibits endogenous gonadotropin secretion, and then leads to the development of secondary sexual characteristics (21, 22).

It is worthy of note that around 37.3% of patients with suprasellar germ cell tumors undergo reactivation or restoration of the HPG axis following treatment, which implies the possible reversibility of HPG axis damage (6). Patients with germ cell tumors may have a more favorable prognosis when treated with radiotherapy and chemotherapy alone. In contrast, other common intracranial tumors in children usually necessitate surgical resection. However, traction and trauma during the surgical procedure might result in irreversible impairment to the HPG axis.

### 3.2 Patients with medulloblastoma

Medulloblastoma is one of the most prevalent malignant pediatric brain tumors, and the common site includes fourth ventricle, cerebellum (23, 24). The median age of onset for medulloblastoma is 7 years old, with a higher incidence in males (25). The impact of medulloblastoma on reproductive function primarily stems from therapeutic interventions such as radiotherapy and chemotherapy (26). Current treatment modalities for medulloblastoma mainly comprise maximal safe resection, accompanied by chemotherapy and radiotherapy based on craniospinal irradiation (CSI) (27).

The damage of medulloblastoma to reproductive function is mainly caused by radiotherapy and chemotherapy, which is more common in female patients, and the recovery of HPG axis function can also be observed (9, 10). The most common reproductive system injury in patients is hypogonadotropic hypogonadism, with precocious puberty being the next in prevalence (13, 26). Studies indicate that the probability of hypogonadism is 23% in patients undergoing CSI, while the probability of precocious puberty is 20% (26). These studies imply that the damage to the HPG axis caused by radiotherapy is the principal factor leading to hypogonadism, and its occurrence rate rises in proportion to higher radiation dosages (12). The mechanism responsible for inducing precocious puberty remains elusive. However, current theories suggest that the damage to inhibitory elements in the hypothalamus results in enhanced signal transduction of gonadotropin-releasing hormone (13, 26).

The long-term adverse reactions of radiation therapy are inversely related to the patient's age at the time of radiotherapy and chemotherapy (8, 9, 12, 25, 28–31). The younger the patients are when they receive radiotherapy, the more severe the adverse reactions will be. Pediatric patients who survive after chemotherapy usually have certain endocrine deficiencies, which can result in gonadal toxicity in 70% of patients (8, 9, 12, 31). Among patients under 5 years old, this proportion increases to 87.5%, especially in female patients (8, 9, 12, 31). Additionally, certain cytotoxic drugs like lomustine, busulfan, and high-dose cisplatin possess inherent gonadotoxic effects (13).

### 3.3 Patients with glioma

Pediatric gliomas originate from glial precursor cells in the brain, and are the most prevalent central nervous system tumors in children, constituting 40%–60% of all pediatric intracranial tumors (32). Gliomas can occur in any brain region and are categorized into low-grade (Grade I and Grade II, comprising 30%–40%) and high-grade (Grade III and Grade IV, comprising 8%–12%) (33). One of the common types of glioma is the H3K27 -altered mutant diffuse midline glioma, which is highly aggressive. Neurosurgeons frequently overlook the patient's reproductive function (34). Nevertheless, in the case of patients with low-grade gliomas, the prognosis is relatively better. Emphasis should be placed on the protection of reproductive function.

When glioma occurs in the vicinity of the pituitary gland and hypothalamus, it can lead to impairment of reproductive function. For example, pediatric optic pathway gliomas (OPG) often give rise to reproductive dysfunction, which presents as diencephalon syndrome. Studies suggest that around 18% of OPG patients have hypothalamic dysfunction, and 20% develop endocrine dysfunction, which may subsequently affect reproductive function, particularly central precocious puberty (CPP) (35–37). When hypothalamic structures are disrupted, gonadotropin-releasing hormone is prematurely pulsatile, leading to early reactivation of the HPG axis and subsequent CPP (38). Moreover, glioma is one of the frequent causes of combined pituitary hormone deficiency (CPHD), and children typically present with symptoms such as hypopituitarism, small or bilateral cryptorchidism (39).

### 3.4 Patients with craniopharyngioma

Craniopharyngioma is a benign tumor, which originates from the craniopharyngeal duct. It is predominantly located in the sellar or parasellar region (40, 41). Craniopharyngioma accounts for 80% of pediatric tumors in the hypothalamic-pituitary region, and the peak incidence occurs between the ages of 5 and 14 years (42, 43). Given its proximity to the hypothalamus and pituitary gland, endocrine and reproductive dysfunctions are frequently observed in pediatric patients (11, 42, 43).

As a result of the direct impairment caused by tumor growth on the hypothalamus and pituitary gland, the majority of patients exhibit symptoms associated with the damage to the HPG axis. Hypogonadism is the most common endocrine abnormality (11). Studies have revealed that approximately 60% of patients exhibit gonadotropin deficiency, resulting in multiple symptoms such as delayed sexual development (42). Endocrine hyperfunction may also present, giving rise to symptoms such as precocious puberty (44).

Various treatment methods for craniopharyngioma can lead to different types of reproductive dysfunction. Patients who undergo surgery are more likely to experience acute and permanent reproductive dysfunction, which is due to the mechanical damage to adjacent tissue structures (45). No phenomenon of HPG axis reactivation or recovery has been observed in patients with craniopharyngioma (4, 5). Instead, the incidence of HPG axis dysfunction may increase (7, 8). Patients who receive radiation therapy may develop late sequelae (45). Given that craniopharyngioma often requires surgical treatment, the damage to the HPG axis during the surgical procedure cannot be overlooked. Recent studies have shown that 75% of children need hormone replacement therapy after the initial surgery, suggesting that surgery has a significant impact on the endocrine system (11).

### 3.5 Patients with pituitary adenomas

Pituitary adenoma, a common benign tumor, accounts for 3% of pediatric intracranial tumors (46). Pituitary adenomas are mainly secretory tumors, with only 5%–10.5% of pediatric cases manifest as non-secreting tumors (46, 47). The impact of pituitary adenomas on reproductive function is mainly due to the tumor's mass effect, which may disrupt the HPG axis, resulting in deficiencies in corresponding hormones. Moreover, specific hormones secreted by the tumor can affect the endocrine system and may consequently affect reproductive function (10, 48).

**Figure 2:**
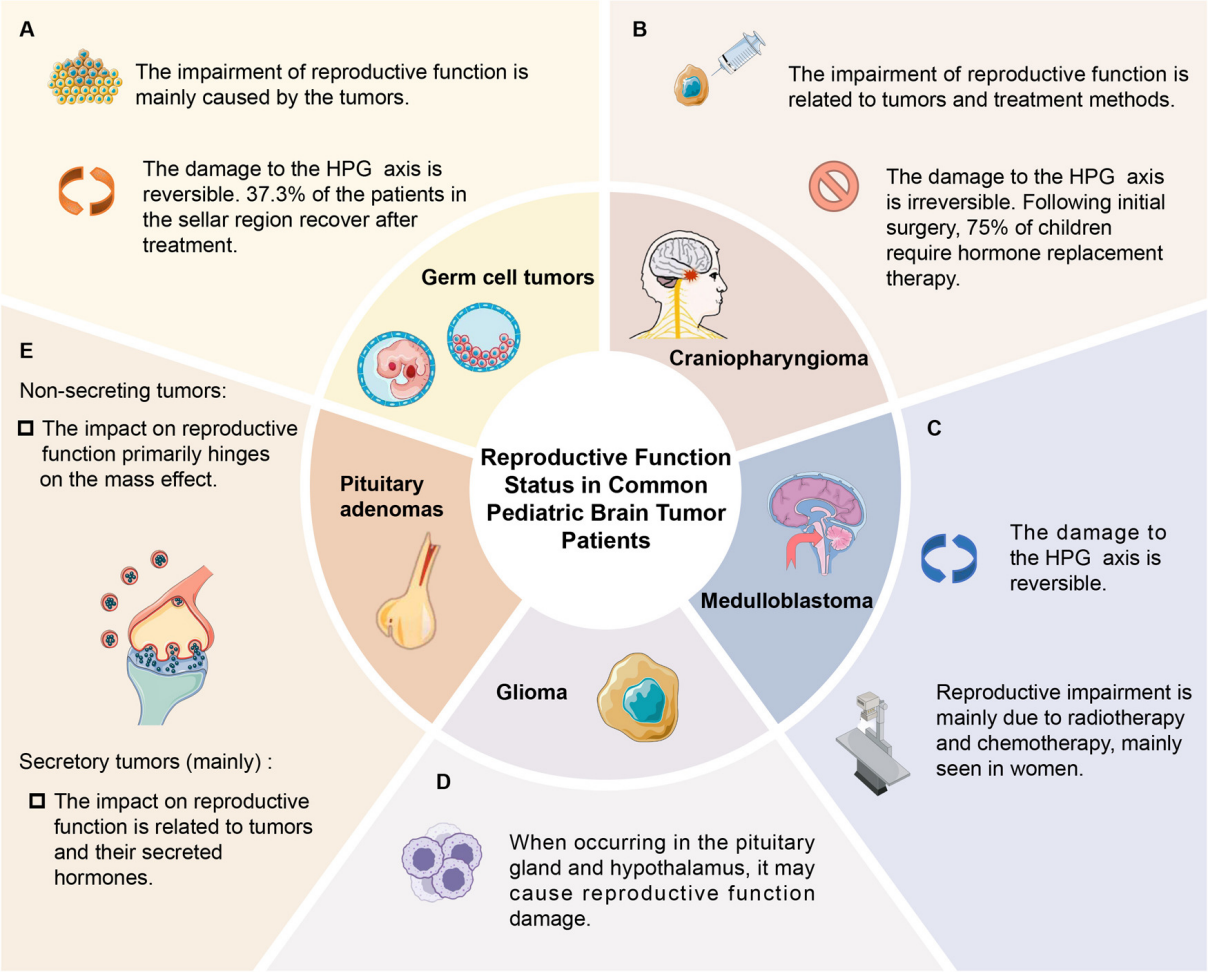
Reproductive function status in pediatric patients with common brain tumors.

Approximately 67% of pituitary adenoma patients develop permanent pituitary insufficiency, which poses a significant threat to the patient's reproductive health (47). Prolactin (PRL)-secreting tumors constitute approximately 53% of pituitary adenomas and are the most prevalent type in children (49). Prolactinomas mainly influence gonadal function via the HPG axis, leading to hypogonadotropic hypogonadism. Patients usually present with symptoms such as hypogonadism and infertility (49). Approximately 75% of female patients present with primary and secondary amenorrhea, while male more commonly exhibit growth arrest and other manifestations (50). Additionally, about 80%–90% patients with non-secreting pituitary adenomas typically present with mass effects such as headache, gonadal dysfunction, subpituitarism, etc. (51). Since the peptides synthesized by gonadotroph adenomas are either not secreted or lack biological activity. In certain cases, however, gonadotroph adenomas may synthesize and secrete biologically active gonadotropins, which could result in some symptoms like central precocious puberty in children (Figure 2) (51–53).

## 4 Protective strategies of reproductive function in pediatric patients with brain tumors

### 4.1 Prophylaxis methods

The impairment of reproductive function in pediatric patients with brain tumors is mainly attributed to the tumors themselves and the treatment modalities. Thus, prevention should initially focus on the risk factors related to these tumors, which can help reduce the incidence rate to a certain extent. Among the numerous potential risk factors identified in epidemiological studies, only ionizing radiation and specific rare genetic syndromes are unambiguously regarded as having a definite etiological impact. The carcinogenic effects of radiation display a distinct dose-response relationship (54). For instance, the cumulative ionizing radiation dose (about 60 mGy) of 2–3 head computed tomography (CT) may triple the risk of brain cancer (55). Although the clinical advantages of CT scans usually prevail over the aforementioned risks, the radiation dose of CT scans ought to be minimized. Additionally, alternative procedures without radiation should be taken into account for the prevention of brain tumors in children. Specific genetic syndromes, such as neurofibromatosis, Li-Fraumeni syndrome, Gorlin syndrome, Turcot syndrome, and hereditary ataxia-telangiectasia, are firmly established factors contributing to the incidence of pediatric brain tumors (56). Furthermore, certain studies have revealed that a family history of malignant brain tumors is also correlated with an elevated risk of brain tumors in children (57). At present, prenatal health evaluations, prenatal screenings, and prenatal diagnostics provide means to avert the birth of children with genetic syndromes.

Although some risk factors are reported in limited studies, we also should attach importance to them. These factors encompass maternal consumption of dietary N-nitroso-compound (NOC), pesticides, smoking, high birth weight, non-chromosomal structural birth defects, fetal growth markers, advanced parental age, etc. (2, 54, 56). What we want to advise is that medical institutions can actively engage in health education targeting preconception populations. This may involve advising prospective parents to minimize or eliminate pickled meat consumption to reduce NOC intake, avoiding exposure to tobacco and pesticides, and undergoing comprehensive health assessments before conceiving.

It's noteworthy that certain researchers conducting epidemiological investigations have identified factors that exhibit a negative correlation with pediatric brain tumors, such as a history of allergies, vitamin, and folic acid supplementation, etc. (56, 58). Studies indicate that a history of allergic diseases may reduce the risk of glioma by 30% (58). The underlying mechanism is associated with the immune enhancement triggered by allergies and the suppression of abnormal brain tumor cell proliferation (58). Similarly, the precise role of vitamin intake and folic acid supplementation remains uncertain, but it may be related to the ability of vitamins to inhibit nitrosation processes, the protective effects of folic acid against the development of neural tube defects (NTDs), and altered enzyme kinetics caused by polymorphisms of folate pathway genes (56, 59).

Other aspects of prevention measures involve the ongoing refinement of treatment strategies for pediatric brain tumors. Treatment modalities typically comprise surgery, radiotherapy, and chemotherapy. Compared with treatment, the changes of reproductive function induced by radiotherapy and chemotherapy are frequently temporary and reversible, while those resulting from surgery tend to be more severe and irreversible. The tumors are situated near the hypothalamus-pituitary gland, and surgical interventions may directly harm the structure of HPG axis, leading to reproductive dysfunction. This highlights the importance of medical workers exercising caution during surgery to mitigate adverse outcomes. Although a consensus on the optimal treatment approach for pediatric craniopharyngioma still has yet to be reached, considering that surgical intervention may exacerbate endocrine dysfunction, the current trend in treatment strategy leans towards conservative approaches to enhance children's long-term quality of life (45, 60). In terms of surgical path, the transsphenoidal technique is more preferred than the previous craniotomy (11). For brain tumors that require radiotherapy and chemotherapy, endeavors should be focused on reducing radiation doses to avert the impairment of the normal function of the HPG axis caused by radiation-induced effects. Moreover, chemotherapy drugs with non-gonadal toxicity should be given precedence to prevent impacts on reproductive function and maintain the overall well-being of children (61). For example, medulloblastoma is usually located far from the hypothalamus and pituitary gland, so the direct damage from surgery is relatively small. The impairment of reproductive function often results from postoperative radiotherapy and chemotherapy. Current research shows that cranial spinal cord radiotherapy (CSRT) with a dose of 18Gy considerably decreases the rate of endocrine morbidity in infants with medulloblastoma compared to conventional doses (62). The balance between therapeutic efficacy and endocrine protection is of great significance and warrants further investigation in the future. In light of the increasing awareness of reproductive dysfunction caused by pediatric brain tumors, international guidelines recommend the proactive evaluation of survivors by endocrinologists (63). Some impairments in reproductive function tend to occur late period (such as in adulthood), so it is necessary for us to formulate transition plans and actively engage in research so as to mitigate the endocrine sequelae of cancer treatment (63). For example, in patients with germ cell tumors, serum prolactin (s-PRL) and thyroid function (TF) assays can be utilized to evaluate the degree of damage to the hypothalamus-pituitar*y* axis and predict the need for post-treatment hormone replacement therapy in children with intracranial pure germ cell tumors (64). This preoperative grading system exhibits higher reliability compared to tumor size alone and promotes the optimization of treatment strategies for childhood germ cell tumors, thereby affording better protection for reproductive function (65). We suggest that more studies can be conducted to predict endocrine function in patients with multiple tumor types to improve treatment guidelines.

### 4.2 Treatment methods

With the advancement of treatments for pediatric brain tumors, reproductive function has increasingly become one of the most prominent concerns among survivors. The current treatment strategies mainly focus on maintaining the normal development of patients' secondary sexual characteristics and their potential for fertility (66). For individuals with central precocious puberty, Gonadotropin-Releasing Hormone analogs (GnRHa) serve as the standard treatment (64, 66). The main aim is to augment adult height and postpone the onset of secondary sexual characteristics to align with those of their peers (64, 66). Traditional Chinese Medicine (TCM) therapy also has a substantial role in the treatment of precocious puberty (64). Treatment regimens for patients with hypogonadism are contingent upon factors like age and disease severity. For instance, in childhood, the focus of treatment is on addressing micropenis and cryptorchidism, whereas hormone replacement therapy proves to be an effective modality in subsequent developmental phases.

To guarantee the relatively high survival rates of pediatric patients with brain tumors, reproductive damage is frequently inevitable. Therefore, fertility preservation (FP) therapy presents itself as a feasible intervention (67, 68). It is essential to consider the indications for fertility preservation. Most current clinical guidelines recommend informing patients who are at risk of fertility damage, interested in preserving fertility, and who may experience reproductive dysfunction after treatment, those undergoing alkylating agent chemotherapy, gonadal radiotherapy, cranial radiotherapy, gonadectomy, or a combination of these treatments, about fertility preservation options (67, 69). In clinical practice, methods for preserving fertility vary according to gender because of the significant disparities in ovarian and testicular function (61). Currently, studies have shown that the options for preserving male fertility are sperm cryopreservation and testicular cryopreservation. In contrast, females have several alternatives, such as cryopreservation of mature oocytes/embryos, ovarian tissue cryopreservation (OTC), ovarian suppression using GnRH agonists, *in vitro* maturation of oocytes, and/or conservative treatment for gynecologic cancers (67, 70–72). For instance, cryopreserved tissue can be transplanted into the autologous ovary or peritoneal cavity, and nearly all cases have demonstrated the recovery of endocrine function in subsequent procedures (73). This strategy is more well-established in the treatment of adult women, yet it is also yielding increasingly favorable outcomes in pediatric patients (Table 1) (71).

**Table 1:** The indications and common techniques for fertility preservation.

Fertility preservation (FP)		
Indications	Before treatment	1. Patients who are at risk of fertility damage
		2. Patients who are interested in preserving fertility
	After treatment (i.e., receiving treatment methods with risks of fertility damage)	1. Alkylating agent chemotherapy
		2. Gonadal radiotherapy
		3. Cranial radiotherapy
		4. Gonadectomy
		5. A combination of above-mentioned treatments
Common techniques	Female	1. Cryopreservation of mature oocytes/embryos
		2. Ovarian tissue cryopreservation (OTC)
		3. Ovarian suppression using GnRH agonists
		4. *In vitro* maturation of oocytes
		5. Conservative treatment for gynecologic cancers
	Male	1. Sperm cryopreservation
		2. Testicular cryopreservation

**Figure 3:**
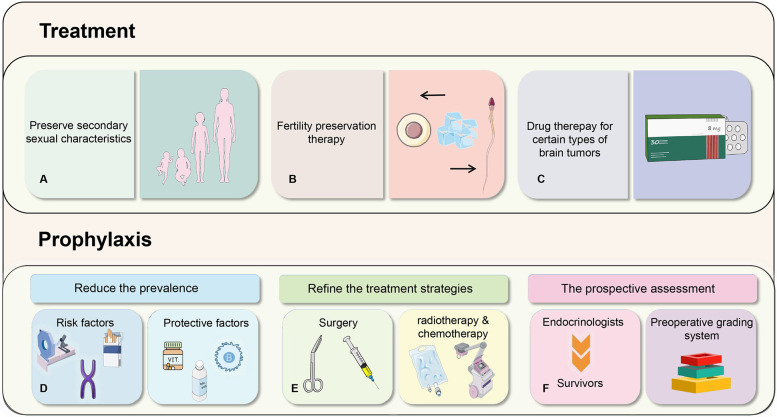
Prophylaxis and treatment methods for reproductive function in pediatric patients with brain tumors.

Furthermore, in the case of certain types of brain tumors, hormone levels can be selectively adjusted to protect reproductive function. For example, in prolactinomas of pituitary adenomas, dopamine-D2 receptor agonist therapy is an effective method to control prolactin levels and preserve gonadal function, as dopamine has the capacity to inhibit prolactin secretion (49). This suggests that more clinical trials of controlling hormone levels should be conducted, which is a promising method for pediatric patients (Figure 3).

## 5 Conclusions

As the most common solid tumors in children, the impairment of pediatric reproductive function is frequent yet often overlooked, which can severely disrupt the normal lives of patients and their families. Reproductive dysfunction is mainly associated with the abnormal HPG axis resulting from the tumor type, location, and treatment modalities. At present, it is crucial to continuously explore the mechanisms underlying reproductive dysfunction caused by brain tumors. Simultaneously, it is essential to formulate a systematic prevention and treatment protocol for maintaining reproductive health in pediatric patients with brain tumors.
